# Control of the upper body accelerations in young and elderly women during level walking

**DOI:** 10.1186/1743-0003-5-30

**Published:** 2008-11-17

**Authors:** Claudia Mazzà, Marco Iosa, Fabrizio Pecoraro, Aurelio Cappozzo

**Affiliations:** 1 Department of Human Movement and Sport Sciences, Università degli Studi di Roma "Foro Italico", Rome, Italy

## Abstract

**Background:**

The control of the head movements during walking allows for the stabilisation of the optic flow, for a more effective processing of the vestibular system signals, and for the consequent control of equilibrium.

In young individuals, the oscillations of the upper body during level walking are characterised by an attenuation of the linear acceleration going from pelvis to head level. In elderly subjects the ability to implement this motor strategy is reduced. The aim of this paper is to go deeper into the mechanisms through which the head accelerations are controlled during level walking, in both young and elderly women specifically.

**Methods:**

A stereophotogrammetric system was used to reconstruct the displacement of markers located at head, shoulder, and pelvis level while 16 young (age: 24 ± 4 years) and 20 older (age: 72 ± 4 years) female volunteers walked at comfortable and fast speed along a linear pathway. The harmonic coefficients of the displacements in the medio-lateral (ML), antero-posterior (AP), and vertical (V) directions were calculated via discrete Fourier transform, and relevant accelerations were computed by analytical double differentiation. The root mean square of the accelerations were used to define three coefficients for quantifying the attenuations of the accelerations from pelvis to head, from pelvis to shoulder, and from shoulder to head.

**Results:**

The coefficients of attenuation were shown to be independent from the walking speed, and hence suitable for group and subject comparison.

The acceleration in the AP direction was attenuated by the two groups both from pelvis to shoulder and from shoulder to head. The reduction of the shoulder to head acceleration, however, was less effective in older women, suggesting that the ability to exploit the cervical hinge to attenuate the AP acceleration is challenged in this population. Young women managed to exploit a pelvis to shoulder attenuation strategy also in the ML direction, whereas in the elderly group the head acceleration was even larger than the pelvis acceleration.

**Conclusion:**

The control of the head acceleration is fundamental when implementing a locomotor strategy and its loss could be one of the causes for walking instability in elderly women.

## Introduction

The oscillations of head, trunk and pelvis during level walking are the result of a compass gait [1]. If seen by an observer moving at the mean velocity of progression, they are characterised by quasi sinusoidal trajectories which, as such, allow for minimal accelerations and, thus, for the stabilisation of the optic flow, for a more effective processing of the vestibular system signals, and for the consequent control of equilibrium [2-4].

In able-bodied individuals, both the lumbar and the cervical hinges play an important role in determining the attenuation of the mechanical perturbations transmitted from the hips, through the pelvis and the spinal column up to the head. This attenuation manifests itself in the fact that the resultant acceleration tends to decrease going from pelvis to head level [5-7]. More specifically, this is mostly due to a decrease of the antero-posterior (AP) acceleration component, as seen through its root mean square (RMS) value. This attenuation has been reported to be already effective at shoulder level [5,8]. The vertical (V) acceleration component displays negligible variations and, as far as the medio-lateral (ML) component is concerned, some authors reported higher RMS values at head than at pelvis level [5], others found no differences between them [9], and some others found lower values at head level [10-12].

The above mentioned results were obtained in volunteer samples either composed of male adults or male and female adults, and gender differences were neither accounted for in the analyses nor investigated. More recently, it has been reported that young females are able to implement a more effective attenuation, possibly indicating a better control strategy [13].

The ability to stabilise the head during walking is expected to be reduced in elderly people due to loss of skeletal muscle strength [14], reduced ability to detect and process proprioceptive information [15] and alterations in the vestibulospinal reflex function [16]. This assumption is corroborated by previous studies, specifically dealing with the control of the upper body accelerations. In fact, it has been reported that whereas young healthy individuals manage to attenuate the accelerations from pelvis to head even when increasing their walking speed [11], this ability is challenged in elderly subjects [9,10]. Furthermore, difficulties in controlling the upper body accelerations have also been reported to be associated with the risk of fall [12].

Nevertheless, there is a controversy in the literature about the amount of attenuation that each acceleration component undergoes. Menz et al. [10] found higher accelerations at head level in the ML direction for the elderly subjects as compared with a control group of young adults, despite smaller accelerations at pelvis level. Kavanagh et al. [9] found significant differences between the two groups only in the AP direction. This discrepancy could be due to the fact that the accelerations were measured at different spine levels (sacrum vs L3), as partially supported by the results of a third study by Marigold and Patla [12], who investigated the ML head to mid-trunk acceleration ratios and found lower values for the elderly than the young subjects. It has to be noted, moreover, that, differently from the other two studies, the study of Kavanagh et al. [9], involved only male subjects. Last but not least, in the latter studies different techniques have been adopted to account for subject anthropometry and walking speed.

The aim of this study is to assess the ability to attenuate the head acceleration during level walking with specific reference to young and elderly individuals. Taking into account the above described possible reasons for the discrepancies found in the literature, this study was limited to female subjects and its aim was pursued by considering three different upper body levels (pelvis, shoulder, and head) and by searching for an index not affected by the strategy chosen by a subject to walk at a certain speed (i.e., typically, the step length and frequency).

## Materials and methods

Sixteen young (young group, YG, age: 24 ± 4 years; height: 1.66 ± 0.05 m; mass: 57.7 ± 7.1 kg) and twenty older (elderly group, EG, age: 72 ± 4 years, height: 1.54 ± 0.06 m, mass: 64.5 ± 7.9 kg) women volunteered for the study and signed an informed consent. All subjects were physically active and had no self-reported musculoskeletal or neurological disorders that could affect their performance and/or behaviour.

A 9-camera VICON MX system (sampling rate = 120 samples/s) was used to reconstruct the trajectories of 8 markers located on the following anatomical landmarks: anterior and posterior superior iliac spines, jugular notch, C7 spinous process, front and back of the head. The measurement volume allowed for the capture of at least one walking stride occurring in the central part of a 12 m long linear pathway. The steady state of the recorded stride was verified using the method presented in [17].

Head, upper trunk, and pelvis movements were described using respectively the trajectories of the midpoint between the head markers (head level), between C7 and the jugular notch (shoulder level), and of the centroid of the iliac spines (pelvis level), which will be referred to as H, S, and P.

A spot check carried out prior to each experimental session [18] showed that the stereophotogrammetric system had an accuracy in the order of 1.4 mm. Soft tissue artefacts were deemed negligible at head and shoulder level. Although no clue about their magnitude was available at pelvis level, these errors were expected to be characterised by a low frequency and negligible power [2].

Subjects were asked to walk at two different self-selected constant speeds of progression described as: comfortable (CS, "walk naturally") and fast (FS, "walk as fast as you can"). Five trials were recorded for each condition.

The stride beginning (t_b_) and ending (t_e_) instants of time were measured using a purposely built instrumented mat [19], where adhesive 5 mm wide copper stripes were attached parallel to each other at a 3 mm distance along a 4 m length linoleum carpet. Alternative stripes were connected to an electric circuit so that, when short circuited, a signal was generated. Two independent circuits were constructed for right and left foot. Subjects wore custom designed socks that hosted a conductive material on their bottom part. The stride period (T = t_e_-t_b_) and frequency (SF = 1/T) were then determined. Stride length (SL) was computed as the antero-posterior displacement of the C7 marker between two sequential heel strikes of the same leg. Walking speed (WS) values were obtained as the product of SL and SF.

The harmonic coefficients of the H, S, and P displacements in the AP, ML, and V directions were then calculated via discrete Fourier transform. The fundamental frequency was set equal to the stride frequency.

The relative power (RP_h_) of each of the harmonics that represent the coordinates in the AP (RP_AP_), ML (RP_ML_), and V (RP_V_) directions at the three upper body levels, was computed using the following equation [19]:

(1) $$RP_{h}=\frac{A_{h}^{2}}{\sum_{h=1}^{N}A_{h}^{2}}\cdot 100$$

where A_*h *_is the amplitude of the *h*-th harmonic and *N *is the total number of the analyzed harmonics (*N *= 10 in this study). The denominator of the equation represents the total power of the *N *harmonics that can be considered as an estimate of the total power of the signal.

Since, in all directions and at all body levels, only the first four harmonics had an amplitude higher than the accuracy of the system and the ratio between the sum of their power, and the total power was higher than 98%, they were the only harmonics used in the further computations.

The accelerations of H, S, and P were then computed by analytical double differentiation of the displacements reconstructed using the first four harmonics. The root mean square of the resultant accelerations at the three levels (RMS_H_, RMS_S_, and RMS_P_) was also calculated.

The harmonic ratio (HR), defined [11] as:

HR = Σ Amplitudes of even harmonics/Σ Amplitudes of odd harmonics

for the AP and V components, and as:

HR = Σ Amplitudes of odd harmonics/Σ Amplitudes of even harmonics

for the ML component, was computed as an indicator of gait rhythmicity with respect to each heel contact. Higher values of HR are associated to a higher similarity between the pattern of the upper body movements occurring during right and left steps.

To quantify the effects of walking speed on acceleration, the magnitude of the correlation between the RMS_H_, RMS_S_, and RMS_P _values and the Froude Number, F_n_, was assessed. F_n _was computed as

(2) $${\text{F}}_{\text{n}}=\frac{{\left(\text{WS}\right)}^{2}}{\text{g}*\text{L}},$$

where g is the gravitational acceleration and *L *is the subject leg length. F_n _was chosen in place of WS since, just like the acceleration data, it depends on the square of the stride frequency. Moreover, F_n _is not affected by the anthropometric characteristics of the subjects.

Finally, to investigate the differences between the two groups in the ability to attenuate the accelerations from pelvis to head level, from pelvis to shoulder level, and from shoulder to head level, the following coefficients were used, respectively:

(3) $${\text{C}}_{\text{PH}}=(1-\frac{{\text{RMS}}_{\text{H}}}{{\text{RMS}}_{\text{P}}})*100,$$

(4) $${\text{C}}_{\text{PS}}=(1-\frac{{\text{RMS}}_{\text{S}}}{{\text{RMS}}_{\text{P}}})*100,$$

and

(5) $${\text{C}}_{\text{SH}}=(1-\frac{{\text{RMS}}_{\text{H}}}{{\text{RMS}}_{\text{S}}})*100,$$

It is important to highlight that these coefficients, being evaluated as a ratio between accelerations, are expected to be independent from the stride frequency of each trial under analysis. Higher values of the coefficients indicate a more effective head stabilisation strategy and a higher reduction of the inertial loads.

### Statistical analysis

The average values of the above parameters were computed for each subject over the different trials. From these values, the sample mean and the standard error of the mean (s.e.m.) were then calculated for the two groups.

To test the overall null hypothesis, a two-way repeated measures analysis of variance (ANOVA) was used. The effects of a within-group factor (condition: two levels, CS and FS) and a between-group factor (age: two levels, YG and EG) on HR, RMS_H_, RMS_S_, and RMS_P_, and C_PH_, C_PS_, and C_SH _were assessed. Since all variables had only two levels, no post-hoc comparisons were performed. However, to separately test the null hypothesis on the differences between YG and EG, planned comparisons were performed at each body level using an unpaired t-test. Similarly, the differences between comfortable and fast speed conditions were assessed using a paired t-test.

A regression analysis and the relevant coefficient of determination (R^2^) were used to assess the dependency of the RMS and of the coefficients C_PH_, C_PS_, and C_SH _on F_n_.

## Results

The YG walked at higher speed and with higher step length than the EG (Table 1). The two groups increased both step length (t-test p < 0.0001) and step frequency (t-test p < 0.0001) when going from CS to FS. The walking speed reached by the YG in the FS trials was significantly higher than that of the EG, but still below the threshold indicating the walking to running transition reported in the literature for young women [20].

**Table 1:** Gait spatio-temporal parameters

	YG		EG	
	CS	FS	CS	FS
WS [ms^-1^]	1.30 (0.07)	2.32 (0.05)	0.97 (0.04)*	1.59 (0.04)*
SL [m]	1.39 (0.04)	1.61 (0.04)	1.14 (0.04)*	1.25 (0.03)*
SF [s^-1^]	0.93 (0.03)	1.47 (0.04)	0.85 (0.02)	1.30 (0.02)*
F_n_	0.20 (0.02)	0.63 (0.03)	0.12 (0.01)*	0.31 (0.01)*

Mean (s.e.m.) values of the spatio-temporal parameters (WS = walking speed; SL = stride length; SF = stride frequency; F_n _= Froude number) for the young (YG) and elderly (EG) groups walking at comfortable (CS) and fast (FS) speed. * = significant difference between the values of the two groups (p < 0.05, t-test).

The changes due to WS in the above reported gait parameters were associated to a change in the task rhythmicity. The ANOVA, in fact, highlighted a significant effect of the condition factor in almost all upper body segments and directions (see Table 2). In particular, as shown by the HR values reported in Table 3, the increase in speed caused a decrease in the gait rhythmicity. In the AP and V directions, this decrease was more marked in the YG (higher values of Δ% in Table 3), whereas the opposite held true in the ML direction. For both groups and in both conditions the HR slightly decreased when going from pelvis to shoulder and even more when going from shoulder to head level. A deeper analysis of the harmonic amplitudes showed that this decrease in the ratio was caused by a reduction of the even harmonics.

**Table 2:** ANOVA of the harmonic ratios

Harmonic Ratio		Age		Condition		Age × Condition	
		F	p	F	p	F	p
AP	H	0.26	0.614	17.39	**< 0.001**	6.12	**0.018**
	S	0.08	0.783	22.58	**< 0.001**	7.99	**0.008**
	P	5.38	**0.026**	3.05	0.090	2.65	0.113

ML	H	3.26	0.080	30.53	**< 0.001**	1.06	0.311
	S	0.08	0.783	22.58	**< 0.001**	7.99	**0.008**
	P	0.01	0.956	22.68	**< 0.001**	1.11	0.300

V	H	6.29	**0.017**	7.59	**0.009**	1.20	0.282
	S	0.46	0.500	12.13	**0.001**	3.46	0.071
	P	4.26	**0.047**	7.57	**0.009**	2.69	0.110

F and p-values resulting from the repeated measures two-way ANOVA performed on the harmonic ratio values computed at the three body levels (H, S, P) in the three directions (AP, ML, V). Age = between subjects factor; Condition = within subject factors. Bold values: p < 0.05.

**Table 3:** Harmonic ratio values

Harmonic Ratio		YG			EG		
		CS	FS	Δ (%)	CS	FS	Δ (%)
AP	H	2.96 (0.40)	1.60 (0.18)	-46%	2.60 (0.21)	2.26 (0.18)	-13%
	S	5.48 (0.70)	2.41 (0.44)	-56%	4.19 (0.33)	3.41 (0.40)	-19%
	P	8.85 (0.79)	6.70 (0.90)	N.S.	6.12 (0.39)	6.05 (0.66)	N.S.

ML	H	3.00 (0.29)	1.78 (0.21)	-41%	3.84 (0.40)	2.06 (0.17)	-46%
	S	3.40 (0.29)	2.17 (0.47)	-36%	4.70 (0.54)	2.40 (0.21)	-49%
	P	2.86 (0.22)	2.04 (0.36)	-29%	3.11 (0.33)	1.82 (0.21)	-42%

V	H	16.34 (1.31)	11.13 (1.79)	-32%	11.26 (1.07)	9.01 (1.39)	-20%
	S	17.57 (1.83)	10.33 (1.65)	-41%	13.72 (1.49)	11.52 (1.72)	-16%
	P	17.04 (1.83)	11.70 (1.56)	-31%	11.60 (0.78)	10.25 (1.59)	-11%

Mean (s.e.m.) values of the Harmonic Ratios computed at each body level (H, S, P) and in each direction (AP, ML, V) for the two groups (YG, CG). When significant, the variation (Δ) of the Harmonic Ratio between the comfortable (CS) and the fast speed (FS) conditions is reported as a percentage of the CS value (t-test, p < 0.05; N.S. = not significant).

**Figure 1:**
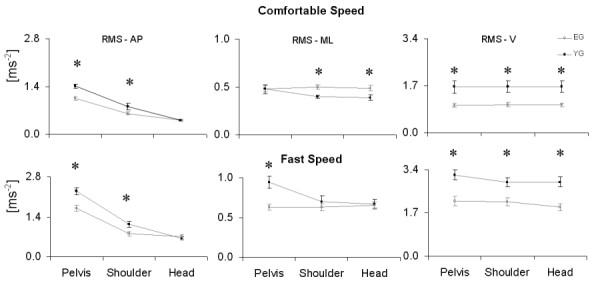
**Acceleration RMS values**. The figure shows the mean ± s.e.m. values of the RMS of the accelerations computed for the two groups at pelvis, shoulder and head level in the two experimental conditions. * = significant difference between YG (black points) and EG (grey points).

The role of the upper body segments in attenuating the oscillations caused by the lower limb movements emerges from the data reported in Figure 1: whereas, as expected, in the V direction the RMS values did not differ among the body levels, in the AP direction they decreased when going from pelvis to head level. This attenuation was present also in the ML direction, but only for the YG (causing the differences between the two groups that were found at shoulder and head level in the CS condition).

The results of the ANOVA performed on the RMS values (Table 4) showed that the effect of the task condition factor was significant at all body levels and in all directions. The effect of the age factor was significant at all levels in the V direction, at pelvis and shoulder level in the AP direction, and only at pelvis level in the ML direction. The interaction between the two factors was found to be significant only in the ML direction at pelvis and shoulder level.

**Table 4:** Analysis of variance on RMS

RMS		Age		Condition		Age × Condition	
		F	p	F	p	F	p
AP	H	0.19	0.667	58.18	**< 0.001**	0.96	0.334
	S	9.71	**0.004**	17.07	**< 0.001**	1.18	0.280
	P	22.88	**< 0.001**	123.17	**< 0.001**	3.37	0.075

ML	H	0.56	0.460	51.38	**< 0.001**	3.27	0.079
	S	0.11	0.741	39.65	**< 0.001**	6.12	**0.019**
	P	7.19	**0.011**	75.23	**< 0.001**	18.85	**< 0.001**

V	H	25.30	**< 0.001**	51.27	**< 0.001**	1.13	0.296
	S	16.46	**< 0.001**	64.56	**< 0.001**	0.15	0.703
	P	22.82	**< 0.001**	57.46	**< 0.001**	1.05	0.313

F and p-values resulting from the repeated measures two-way ANOVA performed on the RMS values of the accelerations computed at the three body levels (H, S, P) in the three directions (AP, ML, V). Age = between subjects factor. Condition = within subject factors. Bold values: p < 0.05.

**Figure 2:**
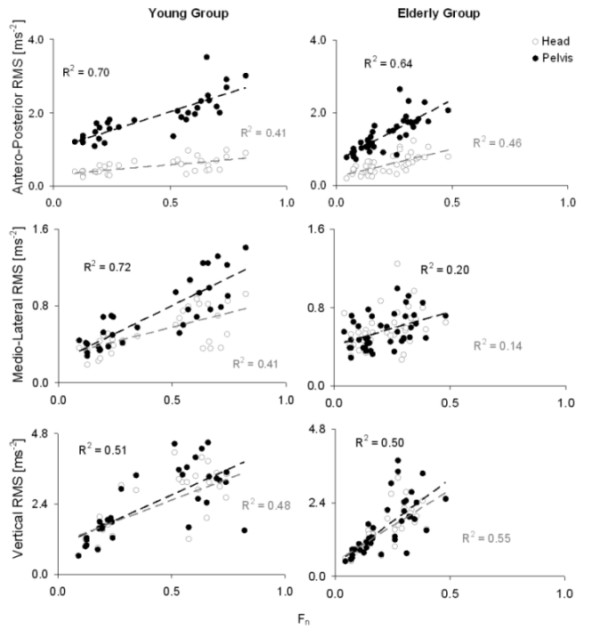
**RMS vs F_n _values**. The figure shows the RMS values (and the relevant linear regression) of the head (light empty circles) and pelvis (dark filled circles) accelerations plotted as a function of the Froude number F_n_.

The results of the regression analysis between the RMS values and the F_n_are illustrated in Figure 2 (where the results relevant to the shoulder have been omitted for the sake of clarity). In the V direction, where no control mechanisms can be put in place by the subjects to attenuate the RMS values, the relationship between these values and the F_n _was the same at head, shoulder, and pelvis level (similar values of the determination coefficient and almost same slope of the relevant regression lines). In the AP direction, the relationship between the RMS and the F_n _was stronger (higher determination coefficients), with a higher slope found at pelvis than at shoulder and head level in the YG but with similar slopes at the three levels found in the EG. In the ML direction, finally, the correlation between RMS and F_n _was still evident in the YG, especially at pelvis level, whereas it was no more significant in the EG.

Very different results were found when the regression analysis with F_n _was performed on the coefficients C_PH_, C_PS_, and C_SH_: no significant correlations were found in the ML and AP direction, and the R^2 ^values were lower than 0.15 indicating that these coefficients are not simply determined by changes in the walking speed. Slightly stronger correlations were found in the V direction, but the R^2 ^values were still quite low (< 0.26).

**Figure 3:**
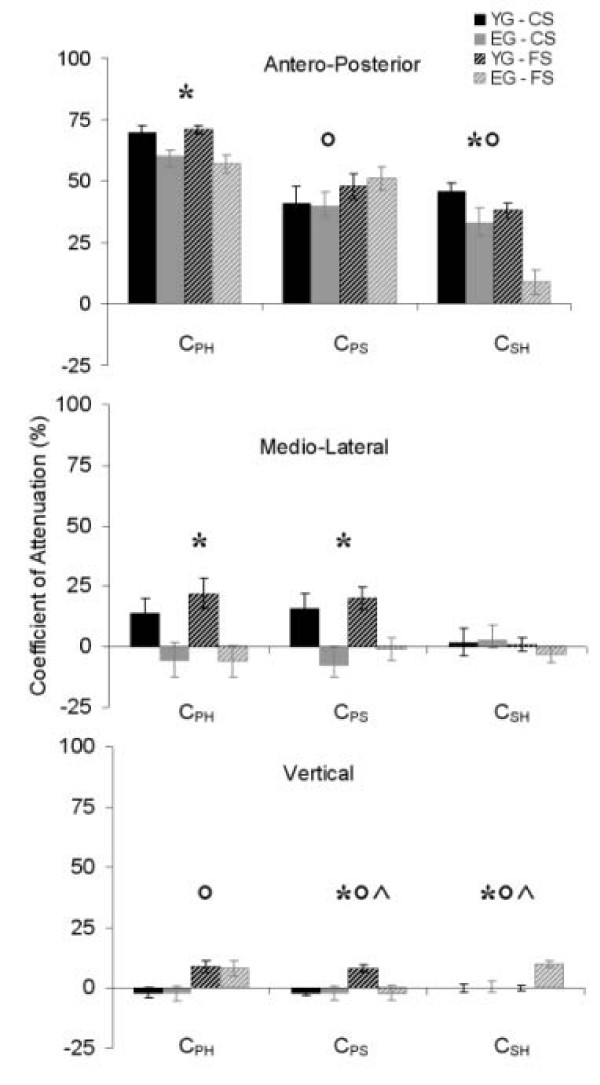
**Coefficients of attenuation**. The figure shows the mean ± s.e.m. values of the three coefficients of attenuation as computed for the young (black bars) and the elderly (grey bars) groups at comfortable (filled bars) and fast (rayed bars) speed. Results of the ANOVA have also been reported: * = age effect; ° = condition effect; ^ = interaction effect; p < 0.05.

The values found for the three coefficients of attenuation, C_PH_, C_PS_, and C_SH_, were very different in the three directions, as appears in Figure 3.

Both groups managed to attenuate the upper body AP accelerations, with an age factor effect (p = 0.007) recorded for C_PH_. This difference between the two groups was mainly due to a difference in the shoulder to head attenuation, which was more effective for the young group (significant effect of the age factor for C_SH_, p < 0.001). The condition factor did not affect C_PH_, but only the other two coefficients.

In the ML direction, not only the elderly subjects did not manage to attenuate the accelerations in the upper body as the YG did, but the accelerations at the head were even increased with respect to those at the pelvis, as shown by the relative negative C_PH _(ANOVA: significant effect of the age factor, p = 0.008). These patterns were due to the fact that, conversely from the YG, no pelvis-shoulder attenuations were found (ANOVA: age factor, p = 0.009). Neither condition nor interaction effects were found.

The low values found for the attenuation coefficients in the V direction reflect the fact that the movements of the upper body segments are strongly coupled due to mechanical constraints. Consistently, the age effect was not significant at pelvis-head level whereas, according to the fact that the speed of the trials could still have affected the coefficients of attenuation results, the condition effect was found to be significant at all levels.

## Discussion

The aim of this paper was to assess differences in the ability of young and elderly women to maintain head stability during waking by controlling the head accelerations. To this purpose, the gait rhythmicity and the rate of the acceleration attenuations have been investigated.

The harmonic ratio has been previously used to assess the rhythmicity of the gait task [10,21] and it has been reported that young healthy adults optimise head stability control by choosing a step length and frequency combination that allows for obtaining the highest HR values in the AP and V direction when walking at the preferred speed, and also in the ML direction when walking at slow speed [11]. Our results (Tables 2 and 3) confirmed this overall pattern for the investigated sample of the young healthy women population, and showed also that in the AP direction the HR values were significantly reduced at head level due to lower amplitudes of the even harmonics. This, on one side, implies a reduction of the frequency content at head level, thus an increased head stability. On the other side, the reduction of the even harmonics also indicates that the head movements become more synchronised with the stride than with the step rhythmicity, suggesting an unexpected loss of symmetry of these movements between the right and the left step, which needs further investigations.

With respect to the HR values, the most evident differences between the two groups were found at pelvis level, where the elderly women had lower AP and V rhythmicity (Table 2). These differences are consistent with the results of Menz et al. [21] who showed that older people with a high risk of falls exhibited less rhythmic acceleration patterns of the pelvis, and can hence be interpreted as a loss of gait stability in our group of elderly women. The HR values were found to diminish for both groups when the subjects were asked to increase their walking speed (Table 3). In the YG, the decrease of HR in the AP and V directions was more marked than in the EG, as a result of the longer strides: a stride length larger than 1.40 m, which in our study occurred only for the YG, in fact, has been reported as the cause of a steep reduction of the AP and V rhythmicity at pelvis level [11].

It has been reported that, during gait, older subjects tend to reduce the pelvic rotations both in the transverse and in the sagittal plane [22]. The analysis of the RMS data reported in this study also showed that the accelerations associated to the pelvis movements were smaller (Figure 1). Moreover, a walking strategy has been highlighted for both young and elderly women aiming at attenuating the ML and the AP accelerations both from pelvis to shoulder and from shoulder to head (Figures 1 and 3).

The reliability of the upper trunk acceleration data has been previously shown to be very high across different experimental conditions, such as slow, preferred, and fast walking speed [23], reflecting the stride-to-stride consistency associated with upper body motion during level walking. Moreover, in healthy elderly women, the harmonic analysis of the upper body movements exhibits both short- and long-term high reliability [19]. It is well known, however, that the outcome measures associated with gait analysis can be strongly affected by changes in preferred walking speed between sessions and subjects and by the subjects' anthropometric characteristics [12,24,25]. The dependence of the acceleration data on the walking speed clearly emerges from the very high coefficients of determination that were found between their RMS and the Froude numbers for our two groups (Figure 2). To overcome this problem, Moe-Nilssen and colleagues [26] suggested a technique for the analysis of the upper body acceleration data, based on the use of a curvilinear interpolation, to compare speed-dependent gait parameters. An optimum use of this method, however, requires the subjects to perform a series of gait tasks in order to obtain data over a representative range of walking speeds. Moreover, it cannot be used to compare gait results acquired during walks with different gait velocities in the same person [25]. The coefficient of attenuation C_PH _proposed in this study to measure the ability of the subjects to control upper body accelerations and preserve head stability, was shown to be independent from the walking speed, and from the task condition in both ML and AP directions. This index is hence suitable for group and subject comparisons.

The importance of the role of the trunk in attenuating AP oscillations has been previously described both for young [9] and elderly [10] subjects, but the mode in which this attenuation mechanism is distributed among the upper body segments has not been fully investigated. Our results showed that the acceleration in the AP direction was attenuated by the two groups both from pelvis to shoulder and from shoulder to head (Figure 3). The reduction of the shoulder to head accelerations, however, appeared more difficult to implement for the older women, especially at fast speed, suggesting that they might have difficulties in using the cervical hinge as an active structure for the attenuation of the AP acceleration. These results somehow confirm what reported by Menz et al. [10], but differ from what more recently reported by Marigold and co. [12]. In the latter study, in fact, no significant difference was found between the two groups in the ratios of the RMS of the head and trunk accelerations, despite this ratio was slightly higher in the elderly subjects. This discrepancy is probably explained by the larger samples involved in our study.

The ML acceleration was more difficult to attenuate than the AP acceleration for both groups (Figure 3), confirming what reported by other authors who investigated the oscillatory dynamics of head and trunk [7]. Whereas the young subjects managed to exploit a pelvis to shoulder attenuation strategy, older ones exhibited head accelerations even higher than the pelvis accelerations. This difference between the two groups could be the consequence of shoulder oscillations needed to accentuate the ML excursion of the whole body centre of mass, a strategy aiming at compensating lower limb muscle weakness. The higher head acceleration may be associated with the difficulty encountered by the elderly group in implementing the neuromuscular control strategies that can help stabilising the postural system in the ML direction during gait. Further studies are needed to test the above hypotheses.

In summary, our results showed that in elderly women the ability to stabilise the head movements during walking is compromised. This ability can be used as an indicator for the assessment of the efficacy of the balance control mechanism. The cause of its limitation could be related not only to muscle weakness but also to a delay of the proprioceptive feedback coming from the trunk and the legs and triggering the movements of the head-neck system, [15] and to alterations in the vestibulocollic reflex function [16]. It might be hypothesised that the latter aspect, associated with the role of the labyrinth, is facilitated by the reduced speed that characterises elderly people walking.

The results obtained in this study using the data measured with a stereophotogrammetric system can be easily reproduced by directly measuring the upper body accelerations. According to the most recent literature, inertial sensors seem to be the best candidates for this application, and their use in conjunction with the coefficients of attenuation is, hence, very promising for a wider clinical use.

## Conclusion

This study showed that the head acceleration is a variable that is kept under control by young healthy women when implementing a locomotor strategy and that the efficacy of this balance control mechanism is compromised in elderly women.

## Competing interests

The authors declare that they have no competing interests.

## Authors' contributions

CM participated in the design of the study and drafted the manuscript. MI participated in the design of the study and performed the computation statistical analysis. FP participated in the experimental sessions and in the data analysis. AC conceived the study and participated in its design and coordination and helped to draft the manuscript. All authors read and approved the final manuscript.
